# Genome‐wide analysis of DNA methylation identifies the apoptosis‐related gene *UQCRH* as a tumor suppressor in renal cancer

**DOI:** 10.1002/1878-0261.13040

**Published:** 2021-07-05

**Authors:** Kosuke Miyakuni, Jun Nishida, Daizo Koinuma, Genta Nagae, Hiroyuki Aburatani, Kohei Miyazono, Shogo Ehata

**Affiliations:** ^1^ Department of Molecular Pathology Graduate School of Medicine The University of Tokyo Japan; ^2^ Genome Science Division Research Center for Advanced Science and Technology The University of Tokyo Japan; ^3^ Environmental Science Center The University of Tokyo Japan

**Keywords:** apoptosis, DNA methylation, renal cancer, UQCRH

## Abstract

DNA hypermethylation is frequently observed in clear cell renal cell carcinoma (ccRCC) and correlates with poor clinical outcomes. However, the detailed function of DNA hypermethylation in ccRCC has not been fully uncovered. Here, we show the role of DNA methylation in ccRCC progression through the identification of a target(s) of DNA methyltransferases (DNMT). Our preclinical model of ccRCC using the serial orthotopic inoculation model showed the upregulation of *DNMT3B* in advanced ccRCC. Pretreatment of advanced ccRCC cells with 5‐aza‐deoxycytidine, a DNMT inhibitor, attenuated the formation of primary tumors through the induction of apoptosis. DNA methylated sites were analyzed genome‐wide using methylation array in reference to RNA‐sequencing data. The gene encoding ubiquinol cytochrome *c* reductase hinge protein (UQCRH), one of the components of mitochondrial complex III, was extracted as a methylation target in advanced ccRCC. Immunohistochemical analysis revealed that the expression of *UQCRH* in human ccRCC tissues was lower than normal adjacent tissues. Silencing of *UQCRH* attenuated the cytochrome *c* release in response to apoptotic stimuli and resulted in enhancement of primary tumor formation *in vivo*, implying the tumor‐suppressive role of UQCRH. Moreover, 5‐aza‐deoxycytidine enhanced the therapeutic efficiency of mammalian target of rapamycin inhibitor everolimus *in vivo*. These findings suggested that the DNMT3B‐induced methylation of *UQCRH* may contribute to renal cancer progression and implicated clinical significance of DNMT inhibitor as a therapeutic option for ccRCC.

## Graphical abstract

DNMT3B‐induced methylation of ubiquinol cytochrome *c* reductase hinge protein (UQCRH) contributes to renal cancer progression. Decreased expression of UQCRH inhibits cyt *c* release from mitochondria, which may be important for anti‐apoptotic phenotype of renal cancer cells. Inhibitors of DNA methyltransferases may enhance the therapeutic efficacy of anticancer drugs.

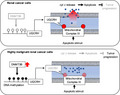

**Abbreviations**
ccRCC: clear cell renal cell carcinoma
DNMT: DNA methyltransferase
mTOR: mammalian target of rapamycin
qRT‐PCR: quantitative RT‐PCR
RNA‐seq: RNA‐sequencing
shRNA: short hairpin RNA
TUNEL: TdT‐mediated dUTP nick end labeling
UQCRH: ubiquinol cytochrome *c* reductase hinge protein

## 1 Introduction

Renal cell carcinoma (RCC) causes more than 100 000 deaths worldwide yearly [1], of which clear cell RCC (ccRCC) is the most common, representing approximately 75% of all adult renal malignancies. The survival rate of ccRCC patients has tremendously improved owing to the advancements in early detection techniques. Patients with localized tumors were treated with nephrectomy. However, regional or distant metastases occur in one‐third of patients with ccRCC. Therefore, the development of new systemic therapies, including molecular target therapy and immunotherapy, is required for metastatic ccRCC.

The inactivation of von Hippel Lindau is frequently observed in ccRCC [2, 3, 4, 5], resulting in impaired ubiquitination and the accumulation of hypoxia‐inducible transcription factor, which induces the expression of various hypoxia‐related genes involved in angiogenesis [6]. Several signaling pathways have also been reported to be activated in ccRCC cells [7]. Based on these findings, molecular target therapies directed toward vascular endothelial growth factor and mammalian target of rapamycin (mTOR) have been developed over the past two decades [8, 9, 10, 11, 12]. However, their therapeutic efficacies remain limited [13, 14].

Other mechanisms have been revealed to play important roles in the ccRCC progression and are thus expected to be potential targets for the treatment of ccRCC. For instance, epigenetic modifications are important not only for carcinogenesis but also for metastasis of ccRCC [15]. Particularly, somatic mutations of genes related to histone modification have been confirmed in ccRCC. Alterations in polybromo 1 (*PBRM1*), breast cancer early onset (*BRCA*)‐associated protein 1 (*BAP1*), SET domain containing 2 (*SETD2*) and enhancer zeste 2 polycomb repressive complex 2 subunit (*EZH2*) are closely associated with clinical outcomes in ccRCC patients [15, 16, 17, 18, 19]. These chromatin regulators affect transcription of a large number of genes, which promote the heterogeneity and evolution of ccRCC cells [20, 21]. We have also previously demonstrated that inflammation‐related signaling is constitutively activated in advanced ccRCC through the formation of a superenhancer [22].

In addition to histone modification, DNA methylation of cytosine is implicated in ccRCC progression [23]. *De novo* DNA methylation is induced mainly at 5′‐C‐phosphate‐G‐3′ (CpG) dinucleotides by DNA methyltransferase (DNMT)3A and DNMT3B, and this methylation process is maintained by DNMT1 [24]. The methylation of DNA cytosine bases leads to the inaccessibility of transcription factors to DNA regulatory elements, which in turn silences the transcription of tumor‐suppressor genes [25, 26, 27, 28]. Recent studies have revealed that DNA hypermethylation is frequently observed in ccRCC and correlates with poor prognosis of ccRCC patients [16]. However, unlike in other cancers, the methylated genes responsible for cancer progression are still unclear in ccRCC. Here, we identified a target of DNMT in ccRCC cells using genome‐wide analysis, and its function in the regulation of cellular survival was evaluated.

## 2 Methods

### 2.1 Cell culture and reagents

Human ccRCC OS‐RC‐2 (RIKEN Cell Bank, Ibaraki, Japan) and their derivatives were cultured in the Roswell Park Memorial Institute 1640 medium (Thermo Fisher Scientific, Waltham, MA, USA) containing 10% FBS (Thermo Fisher Scientific). Human normal proximal tubule HK‐2 cells [American Type Culture Collection (ATCC), Manassas, VA, USA] were cultured in Dulbecco’s modified Eagle’s medium/nutrient mixture F‐12 (DMEM/F‐12) medium (Thermo Fisher Scientific) containing 10% FBS. The HEK293 variant 293FT cells (Thermo Fisher Scientific) were cultured according to the manufacturer’s protocol. Highly malignant derivatives (OS5K‐1, OS5K‐2 and OS5K‐3 cells) were established and maintained as previously described [22]. Cells were authenticated by short tandem repeat analysis. Routine mycoplasma testing was performed. The protein kinase inhibitor staurosporine (Abcam, Cambridge, UK) and the DNMT inhibitor 5‐aza‐deoxycytidine (dC; Sigma‐Aldrich, St. Louis, MO, USA) were reconstituted in DMSO. The caspase inhibitor Z‐VAD‐FMK (G7232) was obtained from Promega Corporation (Madison, WI, USA). The mTOR inhibitor everolimus (RAD001) was obtained from Selleck Chemicals (Houston, TX, USA).

### 2.2 Lentiviral vector construction and production

The lentiviral vector system (provided by H. Miyoshi, deceased, formerly RIKEN) was used for specific gene overexpression and knockdown as previously described [22, 29]. For *UQCRH* overexpression, the cDNA encoding human *UQCRH* was inserted into the multiple cloning site of the empty vector pENTR201. Recombination between pENTR201 and the destination vector CSII‐CMV‐RfA was performed using Gateway Cloning Technology (Thermo Fisher Scientific). The pCSII‐EF‐enhanced GFP was produced as previously described [29]. For UQCRH knockdown, short hairpin RNA (shRNA) targeting UQCRH were inserted into the entry vector pENTR4‐H1. The target sequences for shRNA are shown in Table S1 or as reported previously [30]. Recombination between pENTR4‐H1 and the destination vector pCS‐RfA was performed using Gateway Cloning Technology. The prepared plasmids, pCAG‐HIVgp and pCMV‐VSV‐G‐RSV‐Rev, were transfected into 293FT cells using Lipofectamine 2000 (Thermo Fisher Scientific). The lentiviral vectors were collected from the culture supernatants and concentrated using the Lenti‐X Concentrator (Clontech, Mountain View, CA, USA).

### 2.3 Gene silencing with siRNA

For DNMT3B knockdown, small interfering RNA (siRNA) targeting DNMT3B were used. Cells were plated and transiently transfected with Silencer Select siRNA against DNMT3B (s4221 and s4222; Thermo Fisher Scientific) or Silencer Select Negative Control, Med GC (12935112; Thermo Fisher Scientific) using Lipofectamine RNAiMAX Transfection Reagent (Thermo Fisher Scientific).

### 2.4 Quantitative RT‐PCR analysis

Quantitative RT‐PCR (qRT‐PCR) analysis was performed as previously described [22]. Total RNA was extracted using the ISOGEN Reagent (Nippon Gene, Toyama, Japan) or an RNeasy Mini Kit (Qiagen, Hilden, Germany). The cDNA was synthesized using the PrimeScript II 1st‐stranded cDNA synthesis kit (Takara Bio, Shiga, Japan), and the cDNA products were mixed with FastStart Universal SYBR Green Master Mix with ROX (Roche Diagnostics, Basel, Switzerland) and analyzed using StepOnePlus Real‐Time PCR system (Thermo Fisher Scientific). The expression levels of human *UQCRH* mRNA were normalized to that of human *ACTB* mRNA. The primer sequences are shown in Table S2.

### 2.5 Immunoblotting

Immunoblotting was performed as previously described [22, 31]. Cells were collected, washed with PBS and lysed in RIPA buffer (1% Triton X‐100, 0.5% deoxycholate, 0.1% SDS, 150 mm NaCl and 50 mm Tris/HCl at pH 8.0) containing 1× cOmplete Protease Inhibitor Cocktail (Roche Diagnostics). Cytochrome release was measured using a Cytochrome *c* Release Assay Kit (Abcam) according to the manufacturer’s instructions, subjected to SDS/PAGE, and transferred onto polyvinyl fluoride membranes (Pall Corporation, East Hills, NY, USA) blocked with 5% skim milk containing Tris‐buffered saline with Tween20 (Sigma‐Aldrich; TBST). The membranes were incubated with primary antibodies and the appropriate secondary antibodies (Table S3) diluted in TBST, Can Get Signal 1 (Toyobo, Osaka, Japan) or Can Get Signal 2 (Toyobo). Chemiluminescence images were captured using an ImageQuant LAS 4000 device (Fuji Film, Tokyo, Japan).

### 2.6 Immunohistochemistry and TdT‐mediated dUTP nick end labeling assay

Immunohistochemistry was performed as previously described [22]. For immunostaining, mouse tissues were fixed with Mildform (Wako Pure Chemical, Tokyo, Japan). For human renal tumor tissues, a prefixed human tissue array was purchased (KD2082a, US Biomax Inc., Rockville, MD, USA). After paraffinization, the tissues were sectioned and deparaffinized using xylene and ethanol, followed by antigen retrieval using Universal HIER Antigen Retrieval Reagent (Abcam). Samples were subjected to hematoxylin & eosin staining or immunostaining. After blocking with Block ACE (Bio‐Rad, Hercules, CA, USA), the samples were incubated with primary antibodies and the appropriate secondary antibodies (Table S4) and stained using a Dako Liquid DAB+ Substrate Chromogen System (Agilent Technologies, Santa Clara, CA, USA) and Mayor’s hematoxylin. Images were captured using an AX80 microscope (Olympus, Tokyo, Japan).

To detect apoptosis, *In Situ* Cell Death Detection Kit (TMR red; Roche Diagnostics) and DAPI Fluoromount‐G (Southern Biotech, Birmingham, AL, USA) were used as previously described [32]. Fluorescent images were captured using a BZ‐X710 microscope (KEYENCE, Osaka, Japan).

### 2.7 Immunocytochemistry

Cells were plated on Matsunami Micro Cover Glass (Matsunami, Osaka, Japan), fixed with 4% paraformaldehyde solution and permeabilized with 0.1% Triton X‐100 containing Tween20. Then, they were stained with anticytochrome *c* antibody (12963; 1 : 300, Cell Signaling Technology, Danvers, MA, USA) and visualized using anti‐mouse IgG H&L secondary antibodies (Alexa Fluor 488; Invitrogen, Waltham, MA, USA) in Blocking One reagent (Nacalai Tesque, Kyoto, Japan). The nuclei were stained with DAPI Fluoromount‐G.

### 2.8 Mouse renal orthotopic tumor models

All experiments were approved by the Animal Ethics Committee of the University of Tokyo. The housing and handling conditions of the mice were consistent with the method above. Mouse renal orthotopic tumor models were conducted as previously described [22, 29]. Briefly, BALB/c‐*nu/nu* male mice (5 weeks old) were purchased from Sankyo Labo Service Corporation (Tokyo, Japan). ccRCC cells (1.0 × 10^5^) expressing *Luc2* and mCherry were inoculated into the subrenal capsule of mice. For *in vivo* bioluminescence imaging, D‐luciferin potassium salt (200 mg·kg^−1^; Promega) was diluted in PBS and injected into mice intraperitoneally. For *ex vivo* bioluminescence imaging, the harvested kidneys and lungs were reacted with d‐luciferin potassium solution for 10 min, and images were captured using NightOWL LB981 (Berthold Technologies, Bad Wildbad, Germany). Quantitative analysis was conducted using the IndiGO software (Berthold Technologies). Everolimus was reconstituted in saline solution (Otsuka, Tokyo, Japan) containing 5% Tween20 and 30% propylene glycol (Sigma‐Aldrich) and administered to mice (2.5 mg·kg^−1^) thrice weekly.

### 2.9 Cell proliferation and colony assays

For cell proliferation assay, Cell Counting Kit‐8 (Dojindo Laboratories, Kumamoto, Japan) was used according to the manufacturer’s protocol. The colony formation assay was performed as previously described [33].

### 2.10 Flow cytometry analysis

Flow cytometry analysis was performed as previously described [22]. Briefly, the cells were collected, washed with Annexin V binding buffer (Thermo Fisher Scientific) and reacted with FITC‐conjugated Annexin V (Thermo Fisher Scientific) at room temperature for 10 min. Apoptotic cells were detected using a Gallios flow cytometer (Beckman Coulter, Brea, CA, USA).

### 2.11 Bisulfite‐sequencing analysis

Genomic DNA extraction and bisulfite conversion were performed as described previously [22]. Briefly, genomic DNA was purified using a Gentra Puregene Cell Kit (Qiagen). Bisulfite conversion was performed using an EpiTect Bisulfite Kit (Qiagen). Bisulfited DNA was amplified with Takara Epi‐Taq HS (Takara Bio) using a specific primer for the human *UQCRH* CpG island shore. The primer sequences are listed in Table S5. After ligation with the pCR4‐TOPO vector using the TOPO TA Cloning Kit (Thermo Fisher Scientific), products were transformed into DH5α and sequenced.

### 2.12 Methylation array

Genomic DNA extraction was performed as described previously [22]. A 400‐ng aliquot of genomic DNAs was quantified by Qubit Fluorometer (Life Technologies, Carlsbad, CA, USA) and bisulfite‐converted using an EZ DNA Methylation Kit (Zymo Research, Irvine, CA, USA). Methylation array was conducted using the Infinium Human MethylationEPIC BeadChip Kit (Illumina, San Diego, CA, USA) according to the manufacturer’s protocol. The raw signal intensity for methylated and unmethylated DNA was measured using a BeadArray Scanner (Illumina). After color‐bias correction, background subtraction of the signal intensities and interarray normalization on Genome Studio (Illumina), the raw methylation value (β‐value) for each CpG was defined as M/(M + U + 100), where M and U were the intensities of methylated and unmethylated probes, respectively. CpG loci located 0–500 bp upstream of transcript start sites (TSS) were used for the analysis of methylation status in promoters.

### 2.13 Public database

Data for gene expression and DNA methylation were obtained from public databases: The Cancer Genome Atlas (TCGA) program, Gene Expression Omnibus (GEO; GSE131137, GSE53757 and GSE83820) of the National Center for Biotechnology Information (NCBI) and Cancer Cell Line Encyclopedia of the Broad Institute.

### 2.14 Statistical analysis

Graph generation and statistical analysis were performed using excel (Microsoft, Redmond, WA, USA), JMP pro 14.2, r (v4.0.2, SAS Institute Inc., Cary, NC, USA) and python 3 (Python Software Foundation, Beaverton, OR, USA). No method was used to analyze the sample sizes. Two‐group comparison was performed using Student’s *t*‐test or Welch’s *t*‐test based on the results of the *F*‐test. For multiple comparisons, one‐way analysis of variance (ANOVA), Tukey’s test and Dunnett’s test were used. For the Kaplan–Meier plot analysis, a log‐rank test was used.

## 3 Results

### 3.1 Increased expression of DNMT3B contributes to renal cancer progression

In our previous study [22], orthotopic transplantation was employed to establish highly malignant derivatives of human ccRCC cells (Fig. S1A). Parental OS‐RC‐2 (OSPa) cells were repeatedly exposed to the renal microenvironment. After five serial orthotopic transplantations, three derivatives were obtained as OS5K‐1, OS5K‐2 and OS5K‐3 cells. Although the proliferative ability of OS5K cells did not increase in cell culture (Fig. S1B), the cells exhibited increased tumor formation and metastasis in 3D culture conditions and *in vivo* (Fig. S1C–F), as previously demonstrated [22].

**Fig. 1:**
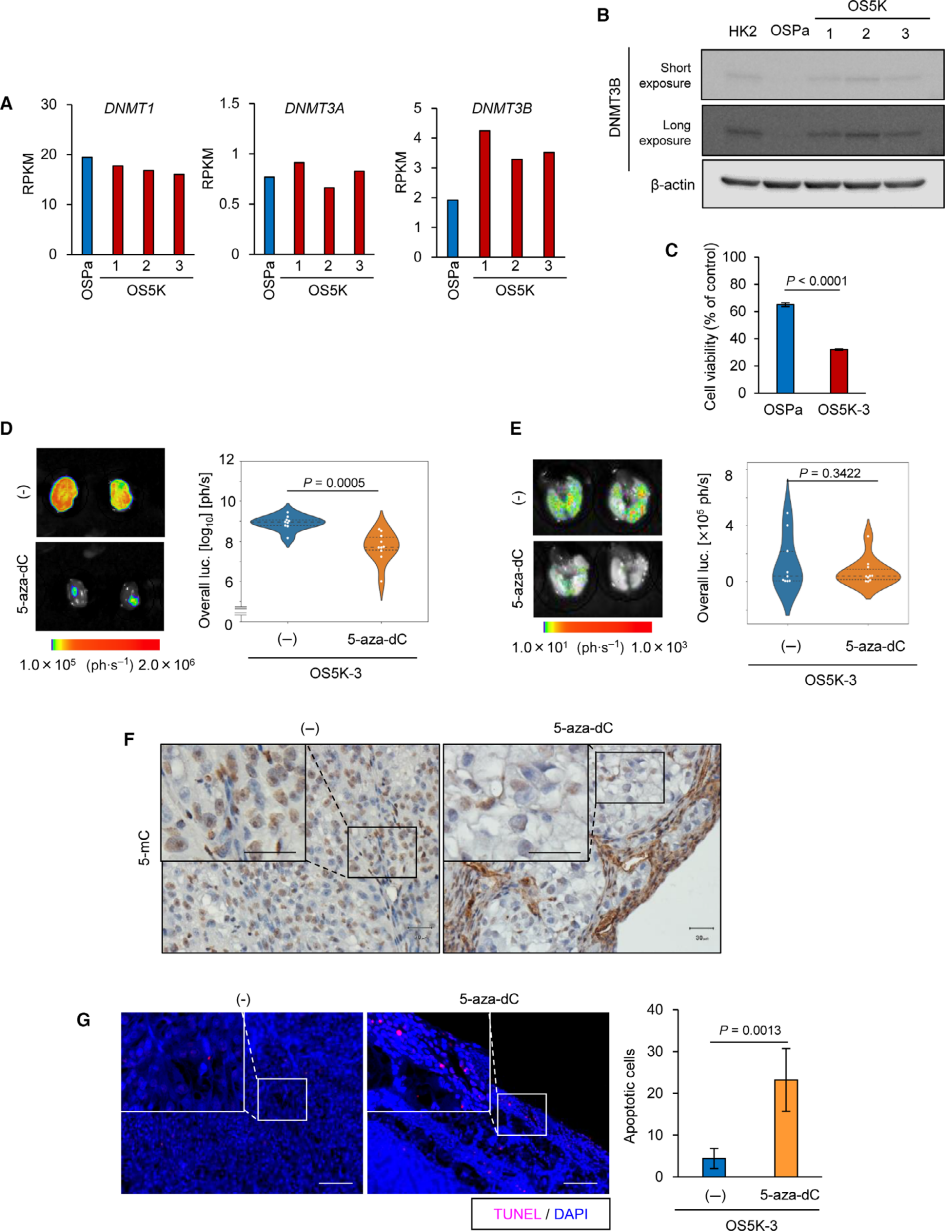
Inhibition of DNMT induces apoptosis of highly malignant ccRCC cells. (A) Expression of DNMT in OS‐RC‐2 derivatives. *DNMT* mRNA were re‐analyzed by RNA‐seq analysis (GSE131137). The expression of DNMT is shown with reads per kilobase of exon per million mapped reads (RPKM) (*n* = 1 sample). (B) Immunoblotting of DNMT3B and β‐actin expression in OS‐RC‐2 derivatives. Representative data from three independent experiments are shown. (C) Cell proliferation assay of OS‐RC‐2 derivatives. The cells were treated with 5‐aza‐dC (1 µm) for 8 days. The percentages compared with untreated control of each cell are indicated. The bars represent the mean ± SD (two‐sided Student’s *t*‐test; *n* = 3 each). (D,E) *Ex vivo* bioluminescence imaging of primary (D) and metastatic lung (E) tumors (left) and quantification (right). OS5K‐3 cells were pretreated with or without 5‐aza‐dC (0.1 µm) for 72 h and inoculated orthotopically in mice. Mice bearing OS‐RC‐2 derivatives were analyzed 17 days after orthotopic inoculation. The bars represent the mean and 1st and 3rd quartiles (D: two‐sided Welch’s *t*‐test; (E) two‐sided Student’s *t*‐test; *n* = 9, control (‐) mice; *n* = 10, 5‐aza‐dC mice). (F) Immunohistochemical staining of the primary tumor tissues in (D) with 5‐methylcytosine (mC). The 5‐mC staining in the boxed region is shown at high magnification. Scale bars: 30 µm. (G) TUNEL assay of the primary tumor tissues in (D) representative images (left) and the number of apoptotic cells in the independent fields (right) are shown. The nucleus was stained with DAPI. Scale bars: 100 µm. The bars represent the mean ± SD (two‐sided Student’s *t*‐test; *n* = 5 each).

To examine the role of DNA methylation during renal cancer progression, the expression levels of DNMT were determined using our previous RNA‐sequencing (RNA‐seq) data (GSE131137) [22] and immunoblotting. The expression of DNMT3B was upregulated in OS5K cells (Fig. 1A,B). We treated these cells with 5‐aza‐dC, a DNMT inhibitor, to diminish the activity of DNMT. Although the viability of OSPa and OS5K cells was decreased by 5‐aza‐dC, 5‐aza‐dC was more potent in suppressing the viability of OS5K cells (Fig. 1C). When xenografted, 5‐aza‐dC pretreatment attenuated the formation of primary tumor in OS5K cells, although lung metastasis was not significantly affected (Fig. 1D,E). Histological examination revealed a decrease in the number of cells with 5‐methylated cytidine in the nuclei, which was accompanied by an increase in TdT‐mediated dUTP nick end labeling (TUNEL)‐positive cells (Fig. 1F,G). These results suggest that DNA methylation is accelerated by DNMT3B in OS5K cells, which may account for their prosurvival phenotype.

**Fig. 2:**
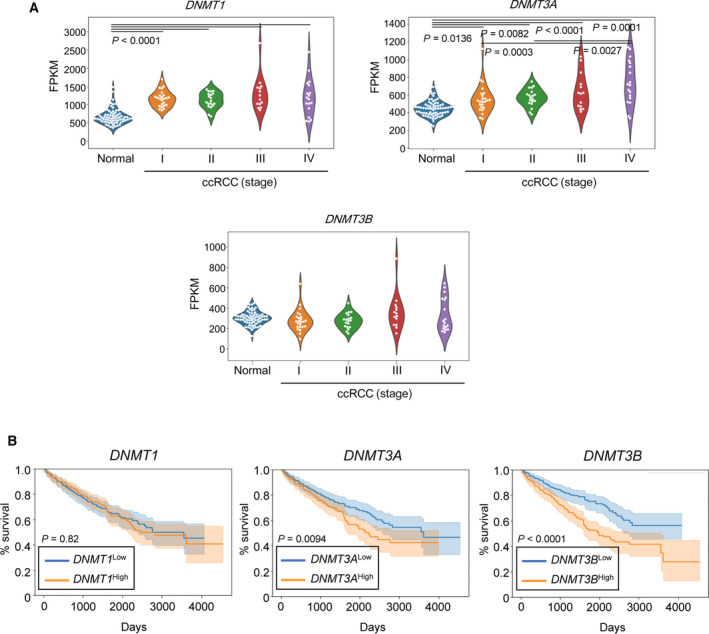
DNMT3B serves as a prognostic marker for renal cancer. (A) Analysis of DNMT expression in ccRCC using the NCBI GEO database (GSE53757). The expression of DNMT is shown with fragments per kilobase of exon per million mapped reads (FPKM) and analyzed using one‐way ANOVA and Tukey’s test (*n* = 69, normal; *n* = 24, ccRCC stage I; *n* = 19, ccRCC stage II; *n* = 14 ccRCC stage III; and *n* = 18, ccRCC stage IV). (B) Correlation between DNMT expression and the overall survival of ccRCC patients. The TCGA database (KIRC) was analyzed and divided into high and low groups of each gene, respectively. Kaplan–Meier plots show the results of applying two prognostic signatures (log‐rank test; *n* = 264 each). Orange lines indicate high groups, and blue lines indicate low groups.

Next, the involvement of DNA methylation in ccRCC progression was confirmed using clinical datasets. In ccRCC cases, increased expression of DNMT1 and DNMT3A, but not of DNMT3B, was observed in a stage‐dependent manner (Fig. 2A). In contrast, poor prognosis of ccRCC patients correlated with the upregulation of DNMT3A and more significantly with DNMT3B (Fig. 2B). Overall, these data suggest that the expression of DNMT3B enhances DNA methylation during ccRCC progression, which may be important in tumor formation and related to poor patient outcomes.

### 3.2 Identification of targets of DNMT3B in renal cancer cells

**Fig. 3:**
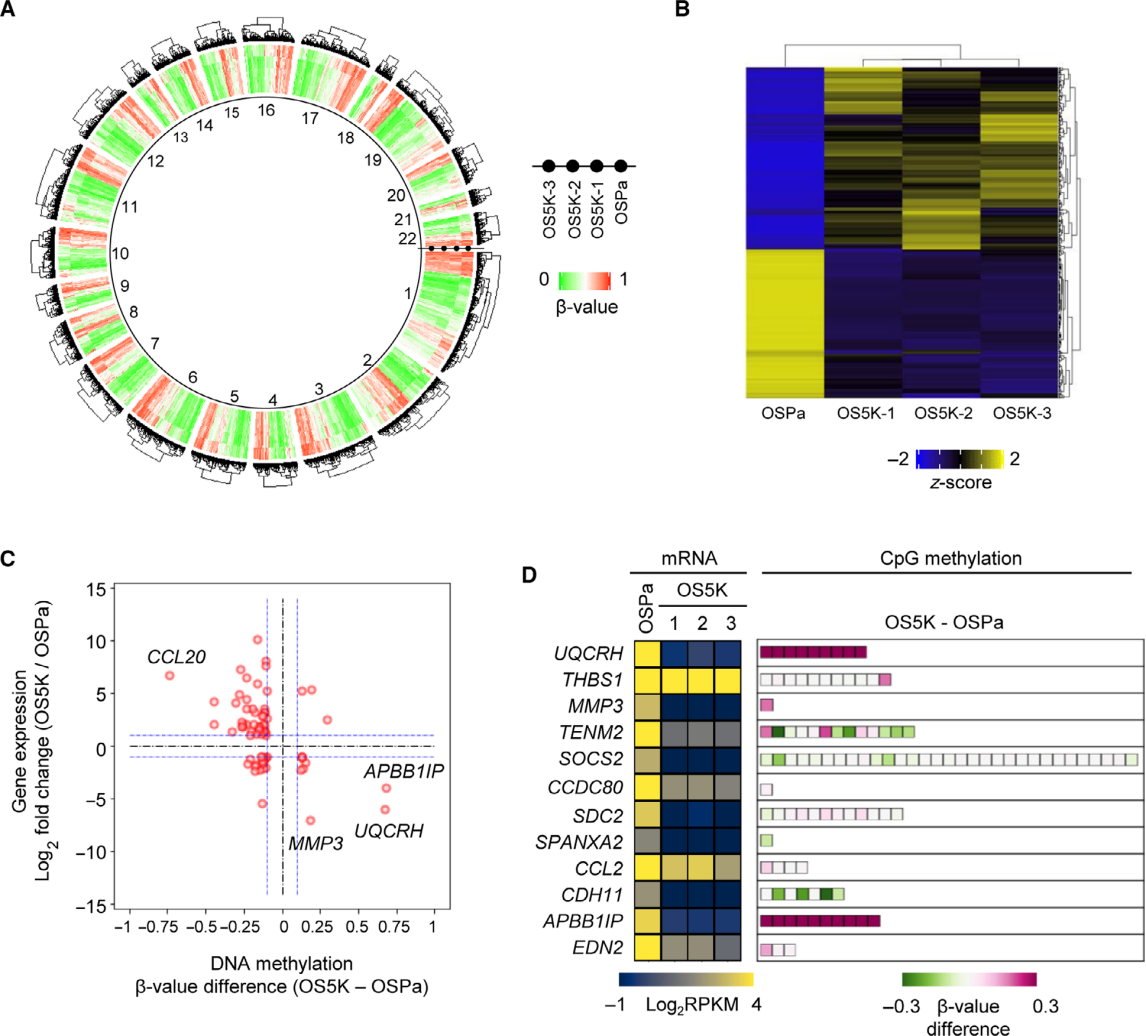
Integrative analysis of ccRCC derivatives using gene expression and DNA methylation in OS‐RC‐2 derivatives. (A) Methylation status as determined by methylation array. Heatmap shows the β‐value of the CpG loci in the promoter DNA in each cell. Clustering was performed on each chromosome (*n* = 2). (B) Gene expression profile using RNA‐seq analysis (GSE131137). Genes whose expression was altered in OS5K cells were screened based on reads per kilobase of exon per million mapped reads [RPKM > 3 in either OSPa or OS5K cells and | Log_2_ (RPKM in OS5K/RPKM in OSPa) | > 1]. The z‐score was then calculated using RPKM in each gene. Each gene expression profile was applied to hierarchical clustering. (*n* = 1). (C) Correlation between DNA methylation and gene expression. Genes were screened by gene expression [RPKM in B, | Log_2_ (RPKM in OS5K/RPKM in OSPa) | > 1] and DNA methylation score (β‐value in A, | β‐value difference | > 0.1). The blue horizontal lines correspond to changes in gene expression more than twice, whereas the blue vertical lines correspond to an absolute difference in β‐value of more than 0.1. (D) Methylation status of the downregulated genes. The significantly decreased genes in OS5K cells were screened and compared with those in OSPa cells in (B) and the methylation status then examined. Boxes represent the probes in their promoter CpG loci in (A). Heatmap shows the gene expression in each cell or the β‐value difference between OS5K and OSPa cells in each promoter CpG locus.

To uncover the role of DNA methylation, genome‐wide screening of DNA methylated sites in ccRCC cells was performed using a methylation array (Fig. 3A). We also re‐analyzed the previous RNA‐seq data (GSE131137; Fig. 3B). We confirmed that the decreased expression of several genes was correlated with DNA methylation (Fig. 3C,D).

**Fig. 4:**
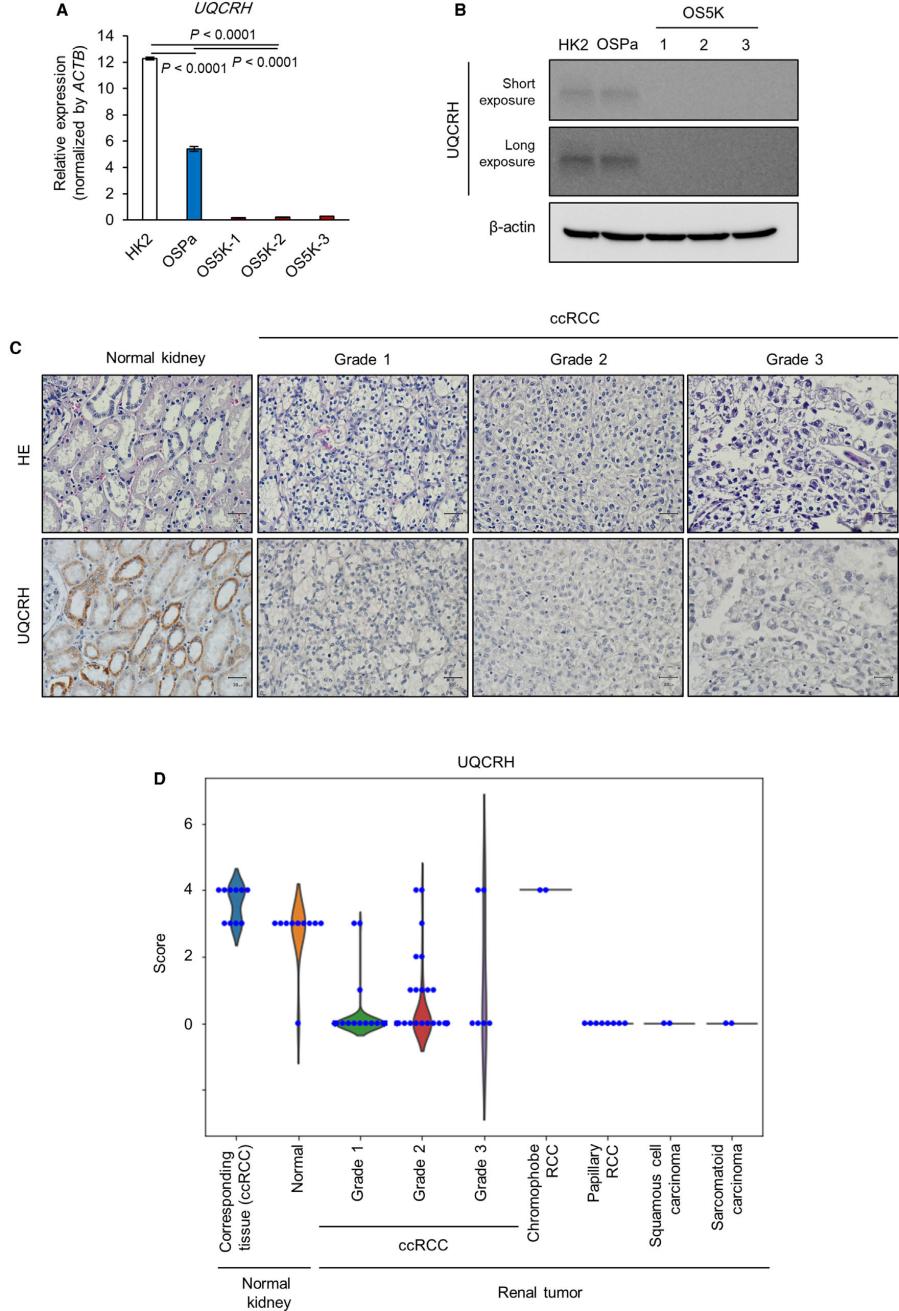
Expression of UQCRH is decreased in human renal tumor tissues. (A,B) Expression of UQCRH in OS‐RC‐2 derivatives and HK‐2 cells (control). *UQCRH* mRNA was analyzed using qRT‐PCR analysis. The bars represent the mean ± SD (one‐way ANOVA and Tukey’s test, *n* = 2). Representative data from three independent experiments are shown (A). Immunoblotting of UQCRH and β‐actin. Representative data from three independent experiments are shown (B). (C,D) Immunohistochemical analysis of human normal renal tissues and renal tumor tissues. Tissue array was stained with HE (top) or anti‐UQCRH antibodies (bottom) (*n* = 96, Grade 1 ccRCC; *n* = 56, Grade 2; *n* = 6, Grade 3; *n* = 10, human normal kidney; *n* = 10, normal adjacent tissue; *n* = 8, papillary RCC; *n* = 2, squamous cell carcinoma; *n* = 2, chromophobe cell carcinoma; and *n* = 2, sarcomatoid carcinoma). Representative images of UQCRH expression in normal kidneys and ccRCC Grades 1–3 (C) and their quantification (D). Scale bar: 30 µm. UQCRH expression in the tissue array was scored as follows: negative = 1; weak = 2; moderate = 3; and strong = 4.

Next, among these methylated genes in OS5K cells, we identified those that may be important for renal cancer progression. Among them, ubiquinol cytochrome *c* reductase hinge protein (*UQCRH*), one of the components of the mitochondrial complex III, was extracted as a methylation target. Compared with normal proximal tubule HK‐2 cells, UQCRH expression was decreased in OSPa cells and further reduced in OS5K cells (Fig. 4A,B). When all of the components of the electron transport chain in OS5K cells were examined, neither gene expression nor methylation status was altered, except for that of UQCRH (Fig. S2).

Clinical database analysis showed that decreased expression of UQCRH was confirmed in cancer cells derived from the kidney and ovary (Fig. S3A). Using the GEO data, we found that the loss of UQCRH was observed during the establishment of patient‐derived xenograft using ccRCC tissues (Fig. S3B). To assess the clinical significance of the decreased expression of UQCRH, its expression in renal tumor tissues was analyzed using clinical samples. Immunohistochemical analysis revealed that the expression of UQCRH in ccRCC tissues was lower than that in normal adjacent tissues, irrespective of the tumor grade (Fig. 4C,D). Moreover, UQCRH expression in other histological types of renal tumor tissues, including papillary RCC, squamous cell carcinoma and sarcomatoid carcinoma, was also lower than that in normal adjacent tissues.

**Fig. 5:**
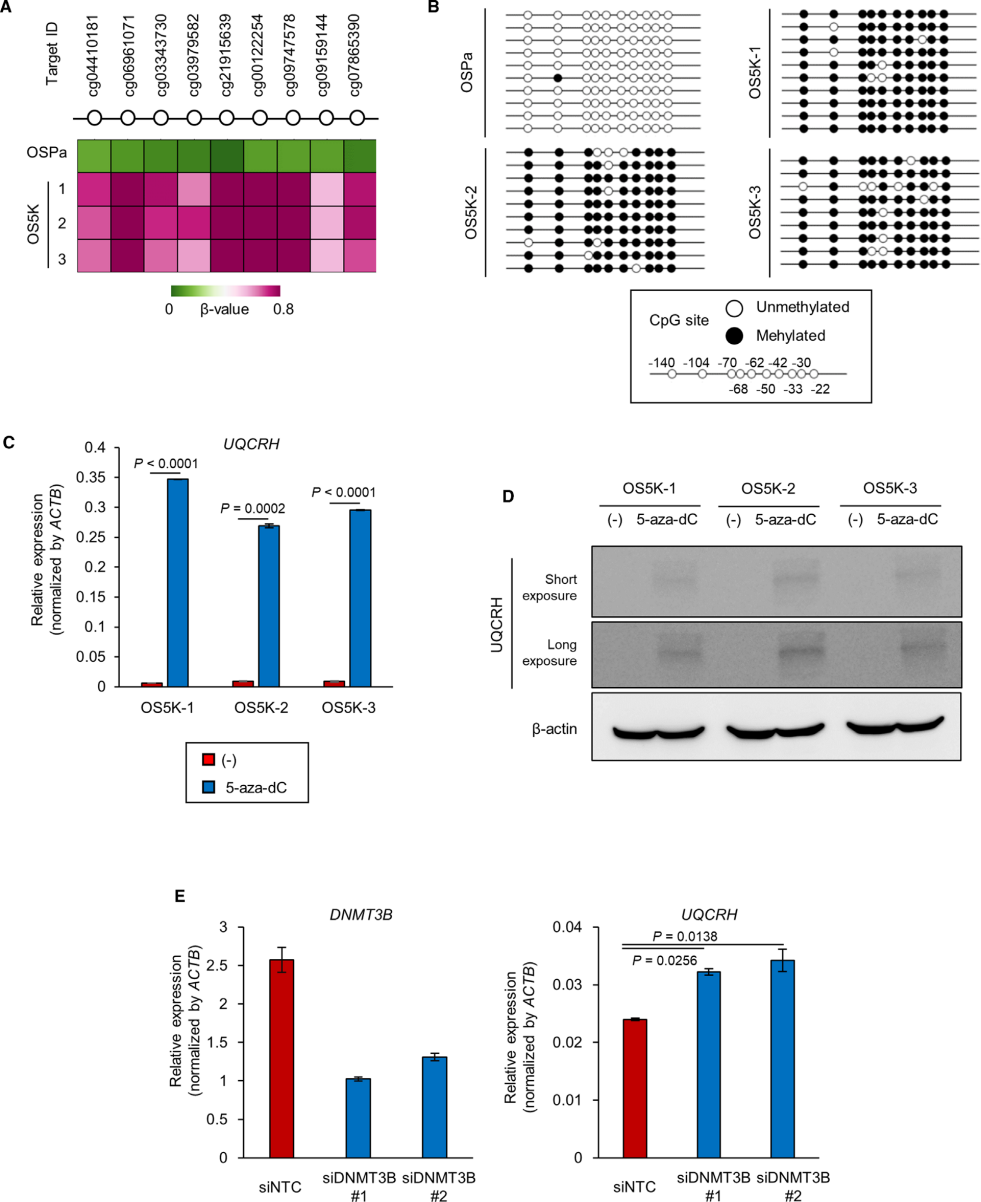
UQCRH expression is downregulated by DNA methylation. (A) Analysis of the methylation status of the *UQCRH* promoter region in OSPa, OS5K‐1, OS5K‐2 and OS5K‐3 cells using the data set from the methylation array in Fig. 3A . Heatmap shows the β‐value. (B) Bisulfite‐sequencing analysis of the *UQCRH* promoter region in OSPa, OS5K‐1, OS5K‐2 and OS5K‐3 cells. Black circles mark the methylated CpG loci; white, unmethylated (*n* = 10, OSPa; *n* = 10, OS5K‐1; *n* = 10, OS5K‐2; and *n* = 9, OS5K‐3). (C,D) Upregulation of UQCRH in OS‐RC‐2 derivatives by the inhibition of DNMT. OS5K cells were cultured with or without 5‐aza‐dC (0.3 µm) for 96 h. *UQCRH* mRNA was analyzed by qRT‐PCR analysis (C). The bars represent the mean ± SD (two‐sided Student’s *t*‐test; *n* = 2). Representative data from three independent experiments are shown. UQCRH protein and β‐actin protein were detected using immunoblotting (D). (E) Upregulation of UQCRH in OS5K‐3 cells by silencing of DNMT3B. OS5K‐3 cells were transfected with control siRNA (siNTC) or siRNA targeting *DNMT3B* (siDNMT3B#1 and #2). After 72 h, gene expression of *DNMT3B* and *UQCRH* was analyzed by qRT‐PCR analysis. The bars represent the mean ± SD (one‐way ANOVA and Dunnett’s test; *n* = 2). Representative data from three independent experiments are shown.

Based on these observations, we focused on the role of UQCRH in ccRCC cells in subsequent experiments. Particularly, the methylation status of the CpG island of the *UQCRH* promoter locus was investigated. The methylation array revealed that all of the CpG loci in *UQCRH* were methylated in OS5K cells but not in OSPa cells (Fig. 5A). Similar results were obtained by bisulfite‐sequencing analysis (Fig. 5B).

Clinical database analysis revealed that the methylation of the promoter in *UQCRH* was frequently observed in cancer cells from the kidney and ovary (Fig. S4A). Notably, the expression of UQCRH was inversely correlated with that of DNMT3B in ccRCC (Fig. S4B).

To directly examine the regulation of UQCRH by DNA methylation, OS5K cells were treated with the DNMT inhibitor 5‐aza‐dC. The expression of UQCRH was restored at both the mRNA and protein levels (Fig. 5C,D). When OS5K cells were transfected with siRNA targeting DNMT3B, the expression of UQCRH was partially recovered (Fig. 5E). These data suggest that the DNMT3B‐mediated decrease in UQCRH may contribute to renal cancer progression.

### 3.3 Renal cancer cells acquire apoptosis resistance through the decrease in UQCRH

**Fig. 6:**
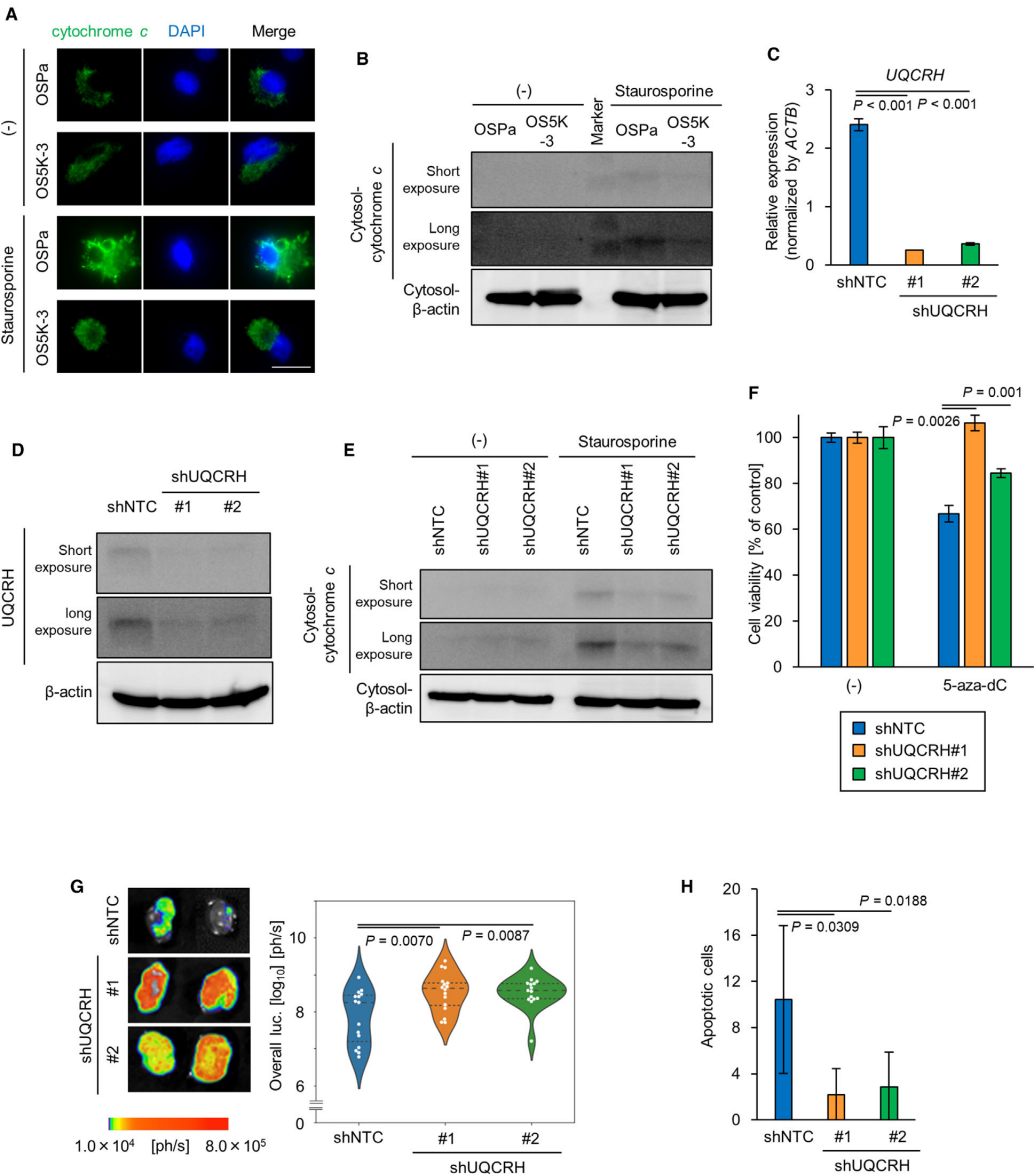
DNMT3B enhances apoptosis resistance by decreasing UQCRH in renal cancer cells. (A) Immunocytochemical staining with cytochrome *c* of OS‐RC‐2 derivatives. Cells were treated with staurosporine (0.5 μm) and Z‐VAD‐FMK (20 µm). Nucleus was stained with DAPI. Scale bars: 25 µm. (B) Immunoblotting of cytochrome *c* and β‐actin in OS‐RC‐2 derivatives. Cells were treated with or without staurosporine (1 µm) for 6 h. Cytosolic fraction was collected. Molecular weight marker (11 and 17 kDa) is indicated. (C,D) Knockdown of UQCRH in OSPa cells. OSPa cells were infected with lentiviral vectors encoding shNTC, shUQCRH#1 or shUQCRH#2. *UQCRH* mRNA was measured using qRT‐PCR analysis (C). The bars represent the mean ± SD (one‐way ANOVA and Tukey’s test, *n* = 2). Representative data from three independent experiments are shown. UQCRH protein was detected using immunoblotting. (D,E) Immunoblotting of cytochrome *c* and β‐actin in ccRCC cells. Cells were treated with staurosporine (1 µm) for 6 h. Cytosolic fraction was collected and subjected to immunoblotting. Representative data from three independent experiments are shown. (F) Cell proliferation assay of ccRCC cells. Cells were cultured with or without 5‐aza‐dC (10 µm) for 96 h. The bars represent the mean ± SD (one‐way ANOVA and Tukey’s test, *n* = 3). Representative data from three independent experiments are shown. (G) Representative images (left) of *ex vivo* bioluminescence imaging of the primary tumors. The ccRCC cells were inoculated orthotopically. The mice were sacrificed 36 days after the inoculation. Panel shows the representative images of primary tumors. Quantification of the luciferase activities from the tumors (right). The bars represent the mean and 1st and 3rd quartiles (one‐way ANOVA and Tukey’s test; *n* = 15, shNTC; *n* = 16, shUQCRH#1; *n* = 15, shUQCRH#2). (H) Apoptotic cells in primary tumor tissues from the experiments in (G). Primary tumor tissues were subjected to TUNEL assay. Nucleus was stained with DAPI. Total apoptotic cells in the independent fields were quantified. The bars represent the mean ± SD (one‐way ANOVA and Tukey’s test; *n* = 7, shNTC; *n* = 6, shUQCRH#1; *n* = 6, shUQCRH#2).

The role of UQCRH expression in apoptosis induction was further investigated. In OSPa cells, the translocation of cytochrome *c* from the mitochondria to the cytosol, an indicator of the initiation of the apoptotic process, was observed following staurosporine treatment (Fig. 6A,B). Subsequently, the cleavage of poly(ADP‐ribose) polymerase (PARP), which indicates caspase activation (Fig. S5A), was observed. However, these apoptotic processes were suppressed in OS5K cells even after treatment with staurosporine.

Next, the involvement of UQCRH in apoptosis induction was examined using overexpression and knockdown experiments. The expression of *UQCRH* in OS5K‐3 cells was recovered using lentiviral vectors (OS5K‐UQCRH cells; Fig. S5B,C). Cytochrome *c* was not released into the cytoplasm in the control OS5K‐GFP cells after staurosporine treatment, whereas it was efficiently released in OS5K‐UQCRH cells (Fig. S5D). We also established UQCRH‐silenced OSPa cells using shRNA (OSPa‐shUQCRH #1, #2 cells; Fig. 6C,D). Immunoblot analysis revealed that the introduction of shUQCRH inhibited the translocation of cytochrome *c* and the cleavage of PARP (Fig. 6E, Fig. S5E). Although 5‐aza‐dC treatment decreased the viability of the control OSPa‐shNTC cells, this effect was partially attenuated in OSPa‐shUQCRH cells (Fig. 6F).

The tumorigenic potentials of OSPa‐shNTC and OSP‐shUQCRH cells were then compared using a mouse renal orthotopic tumor model. When xenografted, OSPa‐shUQCRH cells exhibited significantly faster primary tumor formation than OSPa‐shNTC cells, but lung metastasis was not different in each cell type (Figs 6G and S5F). The number of apoptotic cells was lower in tumor tissues derived from OSPa‐shUQCRH cells than that from OSPa‐shNTC cells (Fig. 6H). These results suggest that UQCRH is essential for the induction of apoptosis and tumor suppression during renal cancer progression.

### 3.4 DNMT inhibitor enhances the therapeutic efficacy of mTOR inhibitor in ccRCC cells

**Fig. 7:**
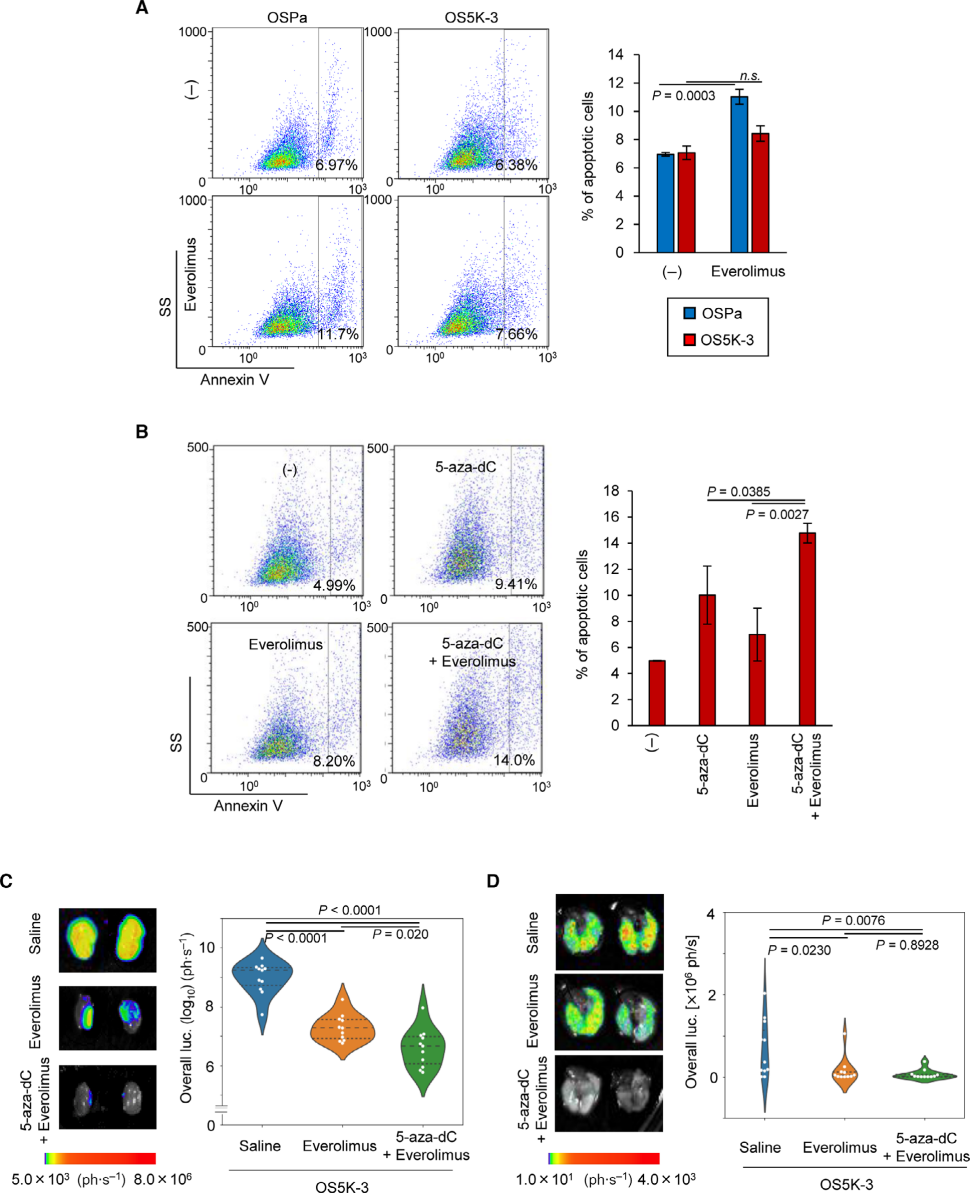
DNMT inhibitor enhances the therapeutic efficacy of mTOR inhibitor in ccRCC cells. (A) Apoptotic cell detection as determined using cytometric analysis of OS5K‐3 cells following everolimus treatment. Cells were treated with or without everolimus (3 µm) for 48 h. Representative panels (left; SS, side scatter); percentages of Annexin V^+^ cell populations (right). The bars represent the mean ± SD (two‐sided Student’s *t*‐test; *n* = 3; n.s, not significant). (B) Apoptotic cell detection as determined using cytometric analysis of OS5K‐3 cells following everolimus or 5‐aza‐dC treatment. Cells were treated with or without everolimus (3 µm) for 24 h or 5‐aza‐dC (0.3 μm) for 72 h. Representative panels (left; SS, side scatter) and percentages of Annexin V^+^ cell populations (right). The bars represent the mean ± SD (one‐way ANOVA and Dunnett’s test; *n* = 3) (C,D) *Ex vivo* bioluminescence imaging of primary (C) and metastatic lung (D) tumors and their quantification. OS5K‐3 cells were pretreated with or without 5‐aza‐dC (0.1 μm) for 72 h and inoculated orthotopically. The mice bearing untreated cancer cells were administrated with saline or everolimus. The mice bearing 5‐aza‐dC‐treated cancer cells were administrated with everolimus. Mice were sacrificed 16 days after the inoculation. The bars represent the mean and 1st and 3rd quartiles (C: one‐way ANOVA and Tukey’s test; D: one‐way ANOVA and Tukey’s test; *n* = 11 each).

Finally, the pharmacological effects of the DNMT inhibitor on drug‐induced apoptosis were examined. Treatment with everolimus, the mTOR inhibitor which is clinically used for patients with renal cancer, successfully inhibited the activation of mTOR and the signaling of its downstream ribosomal protein S6 kinase β‐1 in OSPa and OS5K cells equally (Fig. S6). Nonetheless, everolimus significantly induced apoptosis in OSPa cells and less potently that in OS5K cells (Fig. 7A). When OS5K cells were pretreated with 5‐aza‐dC, the everolimus‐induced apoptosis was enhanced (Fig. 7B).

The combined effect of 5‐aza‐dC and everolimus was further examined *in vivo*. Mice were orthotopically inoculated with OS5K cells, which were treated with 5‐aza‐dC in advance, as shown in Fig. 5. Although 5‐aza‐dC pretreatment did not enhance the effect of everolimus on lung metastasis (at least at the concentration we tested), it significantly augmented the therapeutic effect on primary tumor formation (Fig. 7C,D). These results suggest that DNMT inhibitor may enhance the sensitivity of ccRCC cells to mTOR inhibitors through the recovered expression of UQCRH.

## 4 Discussion

Epigenetic alterations are widely recognized in various human cancer cells [34, 35, 36, 37, 38, 39]. DNA hypermethylation of promoters is induced by the inactivation of DNA demethylases or the overexpression of DNMT [40, 41]. Additionally, mutations in genes encoding metabolism‐related enzymes enhance DNA methylation status. Here, we found the upregulation of DNMT3B in the highly malignant derivatives of ccRCC cells obtained by serial orthotopic inoculations (Fig. 1A,B). Although we have previously established highly metastatic derivatives of pancreatic cancer cells using a similar strategy [42], the expression level of DNMT was not increased in highly metastatic derivatives of pancreatic cancer cells (GSE107960), suggesting that the interactions between cancer cells and the renal microenvironment may be crucial for the upregulation of DNMT3B. Several regulatory mechanisms are speculated to be involved in the increased expression of DNMT3 in cancer cells. For instance, interleukin (IL)‐6 induced the expression of DNMT3B in a signal transducer and the activator of transcription (STAT)3‐dependent manner, resulting in the accelerated proliferation of ccRCC cells or oral squamous cell carcinoma cells [43, 44]. Similarly, here, RNA‐seq results revealed the enrichment of hallmarks of IL‐6–Janus kinase–STAT3 signaling in OS5Ks (K. Miyakuni, unpubl. data). The regulation of DNMT by microRNA has also been described in various cancer cells [45]. Although miR4465 and miR‐29c‐3p may regulate the expression of DNMT3B [46, 47], this was not evident in OS5K cells (K. Miyakuni, unpubl. data).

In addition, recent studies have identified many targets for DNMT3B, including tumor‐suppressor genes in cancer cells [48]. Colorectal cancer, one of the most common DNA methylated cancers, exhibits decreased expression of the cyclin‐dependent kinase inhibitor 2A (*CDKN2A*) and Ras association (RalGDS/AF‐6) domain family member 1 (*RASSF1A*) [49, 50]. Cadherin 1 (*CDH1*), *CDKN2A*, the runt‐related transcription factor 3 (*RUNX3*), *BRCA1* and *RASSF1A* are reported to be suppressed by DNMT3B‐dependent DNA hypermethylation in gastric or breast cancers [51, 52]. In the present study, we conducted genome‐wide screening of methylation targets in ccRCC cells using RNA‐seq analysis and methylation array. Amyloid beta A4 precursor protein‐binding, family B, member 1 interacting protein (APBB1IP) was extracted as a gene whose expression was epigenetically decreased in highly malignant derivatives in ccRCC cells. The knockdown of APBB1IP promoted apoptosis in ccRCC cells [53], which could not account for the anti‐apoptotic phenotype of highly malignant derivatives we obtained. On the other hand, decreased expression of UQCRH is in accordance with the results obtained using clinical ccRCC samples in other cohorts [16], indicating that the downregulation of UQCRH is common in ccRCC. The expression of UQCRH is similarly decreased in several types of cancers [54]. Among them, we found decreased expression of UQCRH in clear cell carcinoma of ovarian cancer (Fig. S3A). The histological phenotype of clear cell carcinoma is likely to be associated with the downregulation of UQCRH, irrespective of the origin of cancers.

UQCRH is highly conserved in many species and serves as a component of the mitochondrial complex III. It is closely associated with the function of cytochrome *c* and cytochrome *c1* [55, 56, 57]. Although UQCRH is also involved in electron transport and the maturation of cytochrome *c1* [58, 59, 60], its role in cancer progression is controversial. Based on histological examination, elevated expression of UQCRH was observed in breast cancer and hepatocellular carcinoma [61, 62]. In contrast, decreased expression of UQCRH was observed in RCC [63]. Although its significance in the Warburg effect was documented in RCC [64], metabolic activities were not clearly different between the parental cells and derivatives in our model (data not shown). Since the introduction of UQCRH was shown to induce apoptotic cell death of certain types of cells [65], we investigated whether the UQCRH regulates the apoptosis of renal cancer cells. In the absence of an apoptotic stimuli, cytochrome *c* is anchored to the inner mitochondrial membrane by binding to cardiolipin. This binding is attenuated when the inner mitochondrial membrane is oxidized by the accumulation of reactive oxygen species (ROS), resulting in the release of cytochrome *c* [66, 67, 68]. Since UQCRH regulates the production of ROS [58], we hypothesized that UQCRH may be essential for the induction of apoptosis in cancer cells. Accordingly, we showed that the silencing of UQCRH attenuated the translocation of cytochrome *c* and the apoptosis in ccRCC cells treated with staurosporine (Figs 6 and S5). These findings suggest that UQCRH may serve as a potential tumor suppressor in ccRCC through the regulation of apoptosis.

DNMT inhibitors, namely 5‐azacytidine and 5‐aza‐2′‐dC, were initially considered for cancer treatment. However, due to their toxicity, only a low dose is recommended. A clinical trial of DNMT inhibitors in combination with other anticancer drugs, such as IL‐2, interferon‐α and bevacizumab, for renal cancer treatment has been conducted [23]. Here, our results demonstrated that silencing of UQCRH enhances the drug‐induced apoptosis in ccRCC cells (Fig. 6) and that the 5‐aza‐dC treatment sensitizes the antitumor effect of mTOR inhibitor both *in vivo* and *in vitro* (Fig. 7). Our results contribute to further understanding of the molecular mechanisms involved in the drug resistance of ccRCC cells and provide an important insight into therapeutic options for ccRCC.

## 5 Conclusions

Here, we report the tumor progressive role of DNMT3B in ccRCC. Our results suggest that DNA methylation causes the decreased expression of the potential tumor‐suppressor gene *UQCRH*, which is essential for the completion of the apoptotic process in ccRCC cells. In our preclinical study, DNA demethylation induced by 5‐aza‐dC enhanced the tumor‐suppressive ability of everolimus. These findings confirm the clinical potential of DNMT inhibitors for ccRCC treatment.

## Conflict of interest

K. Miyazono and SE were partly supported by Eisai, Co., Ltd. The remaining authors declare no conflicts of interest.

## Author contributions

K Miyakuni and SE conceived the study. K Miyakuni and JN performed most of the experiments. DK, GN, and HA assisted in the methylation array. K Miyazono and SE supervised the project and wrote the manuscript. All authors discussed the results and commented on the manuscript.

### Peer Review

The peer review history for this article is available at https://publons.com/publon/10.1002/1878‐0261.13040.

## Supporting information


**Fig. S1**. Establishment of ccRCC derivatives using serial orthotopic transplantation model.

**Fig. S2**. Expression and methylation status of genes encoding electron transport chain components in OS‐RC‐2 derivatives.

**Fig. S3**. Clinical significance of UQCRH downregulation in human cancers.

**Fig. S4**. Correlation between DNA methylation and UQCRH expression in human cancers.

**Fig. S5**. UQCRH regulates the induction of apoptosis in ccRCC cells.

**Fig. S6**. Inhibition of mTOR signaling in OS‐RC‐2 derivatives by everolimus.

**Table S1**. Target sequences for shRNA.
**Table S2**. Prime sequences for qRT‐PCR analysis.
**Table S3**. Antibodies for immunoblotting.
**Table S4**. Antibodies for immunohistochemistry.
**Table S5**. Primer sequences for bisulfite‐sequencing analysis.

## Data Availability

Raw and processed RNA‐seq data are available at GEO. The additional data that support the findings of this study are available from the corresponding author upon reasonable request.
