# Supplementary figures and images for: Genome‐wide analysis of DNA methylation identifies the apoptosis‐related gene UQCRH as a tumor suppressor in renal cancer
Source: Mol Oncol. 2021 Jul 5;16(3):732–49. doi: 10.1002/1878-0261.13040 (PMC8807364; doi:10.1002/1878-0261.13040)

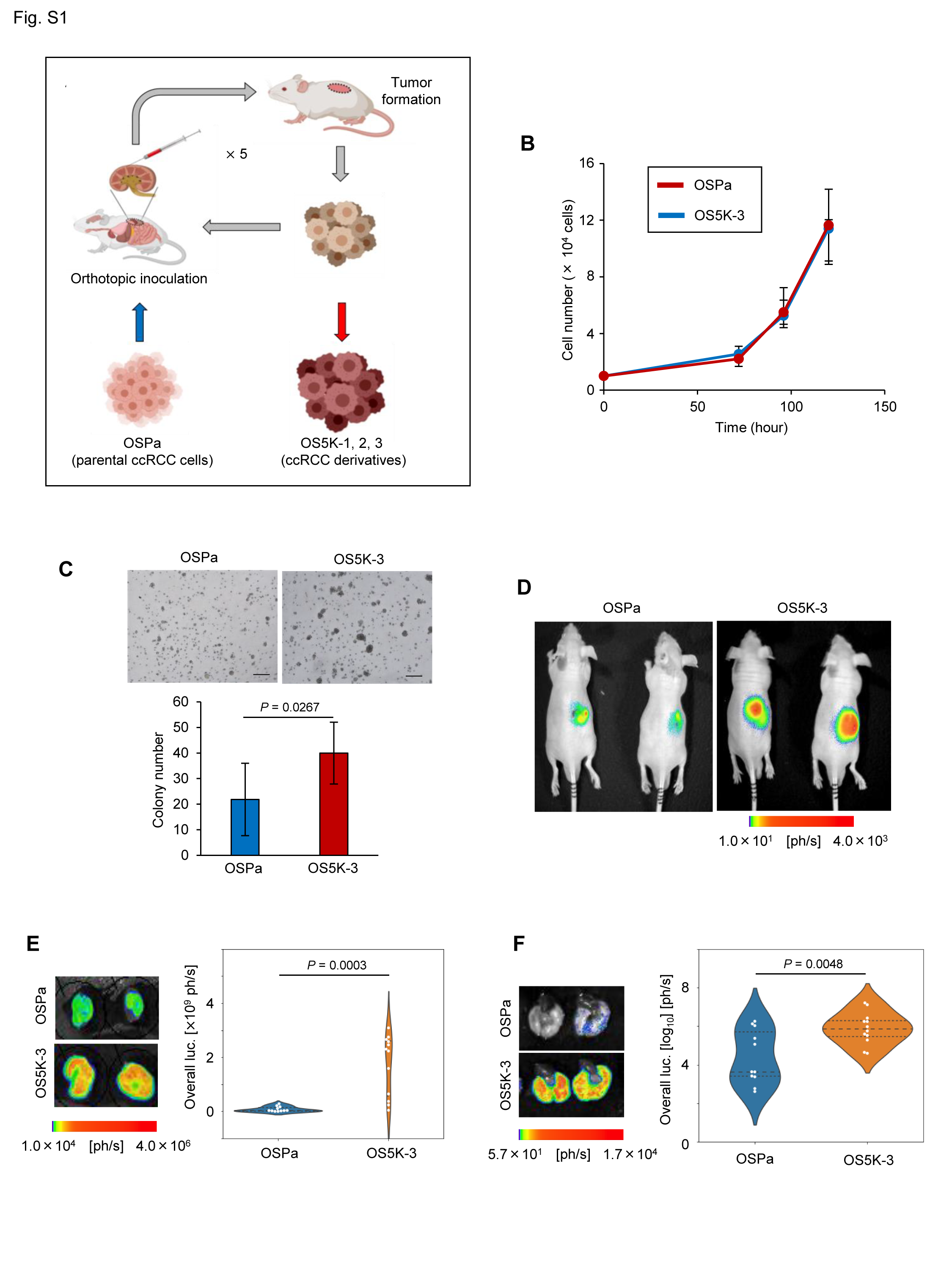

Supplement: Supplementary file 1 — Fig. S1. Establishment of ccRCC derivatives using serial orthotopic transplantation model. [file MOL2-16-732-s004.tif]

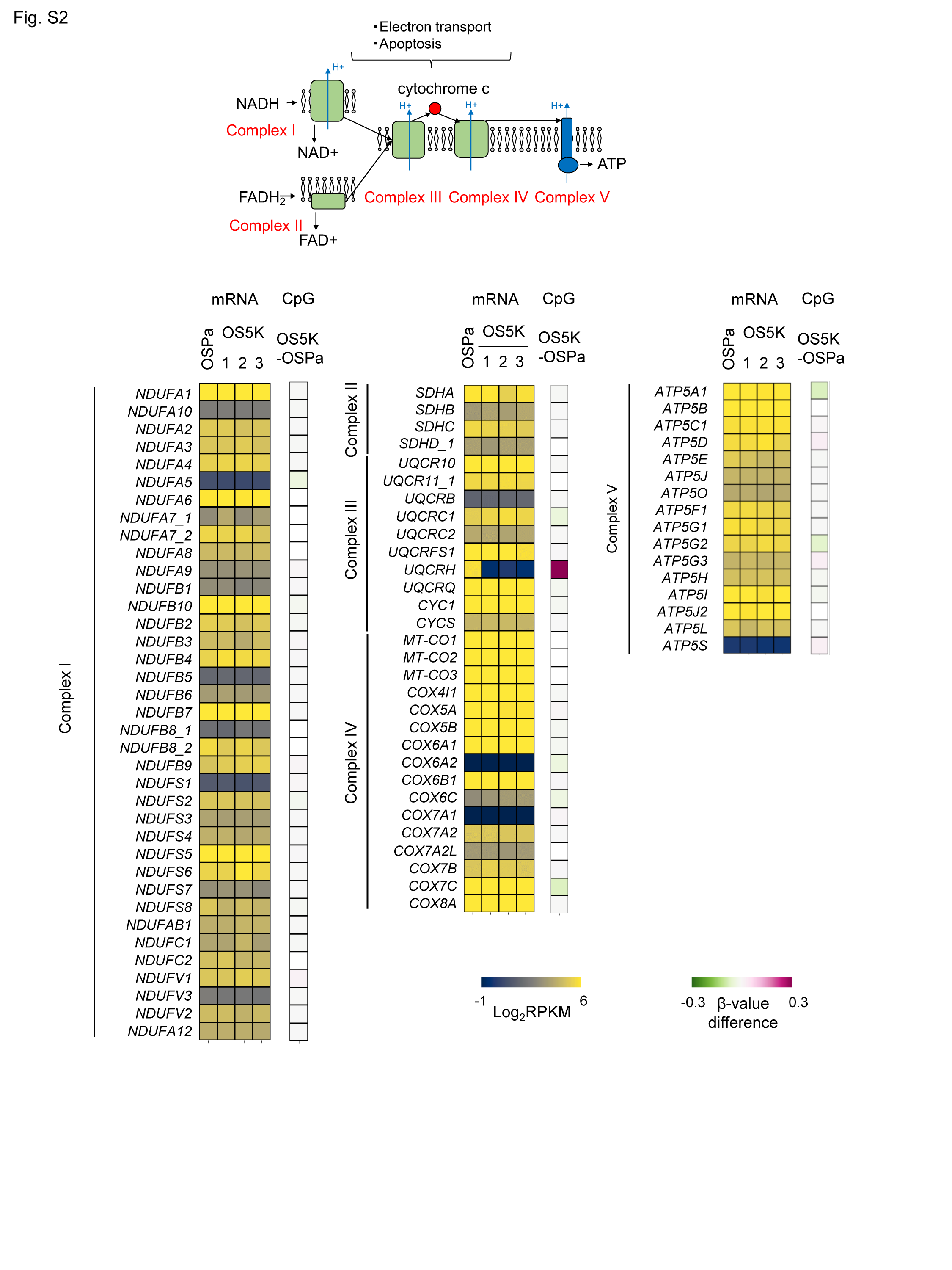

Supplement: Supplementary file 2 — Fig. S2. Expression and methylation status of genes encoding electron transport chain components in OS‐RC‐2 derivatives. [file MOL2-16-732-s002.tif]

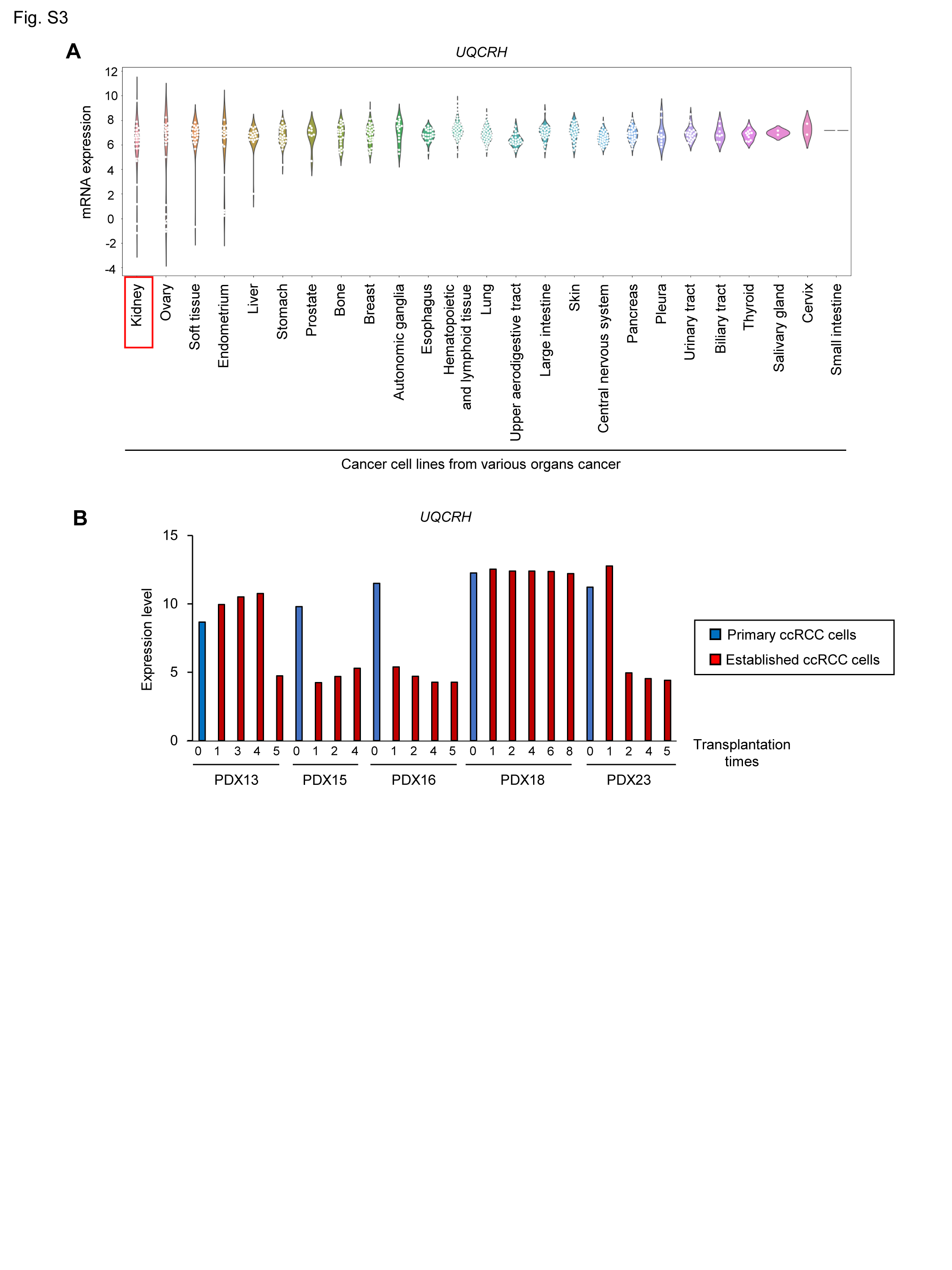

Supplement: Supplementary file 3 — Fig. S3. Clinical significance of UQCRH downregulation in human cancers. [file MOL2-16-732-s003.tif]

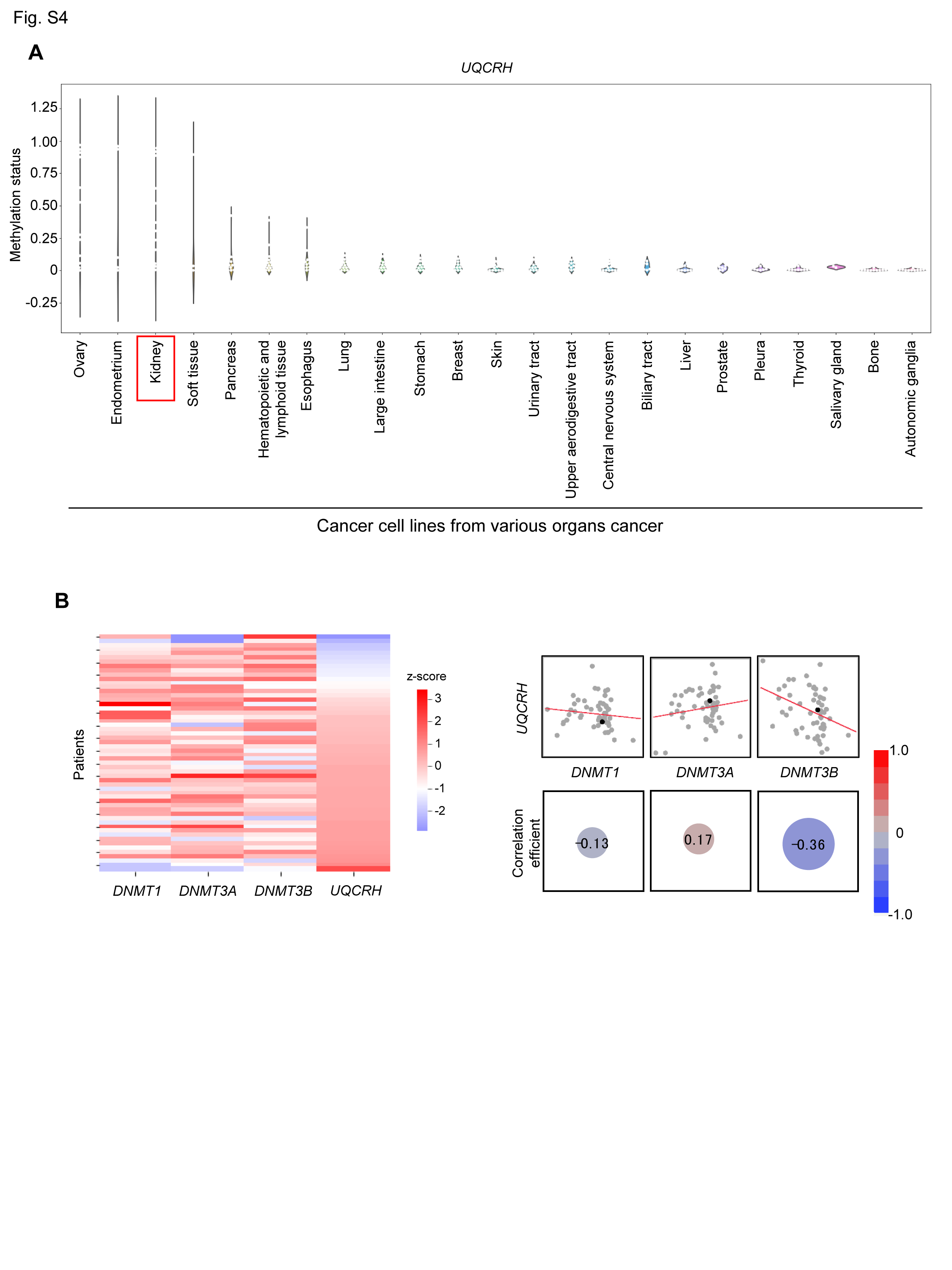

Supplement: Supplementary file 4 — Fig. S4. Correlation between DNA methylation and UQCRH expression in human cancers. [file MOL2-16-732-s006.tif]

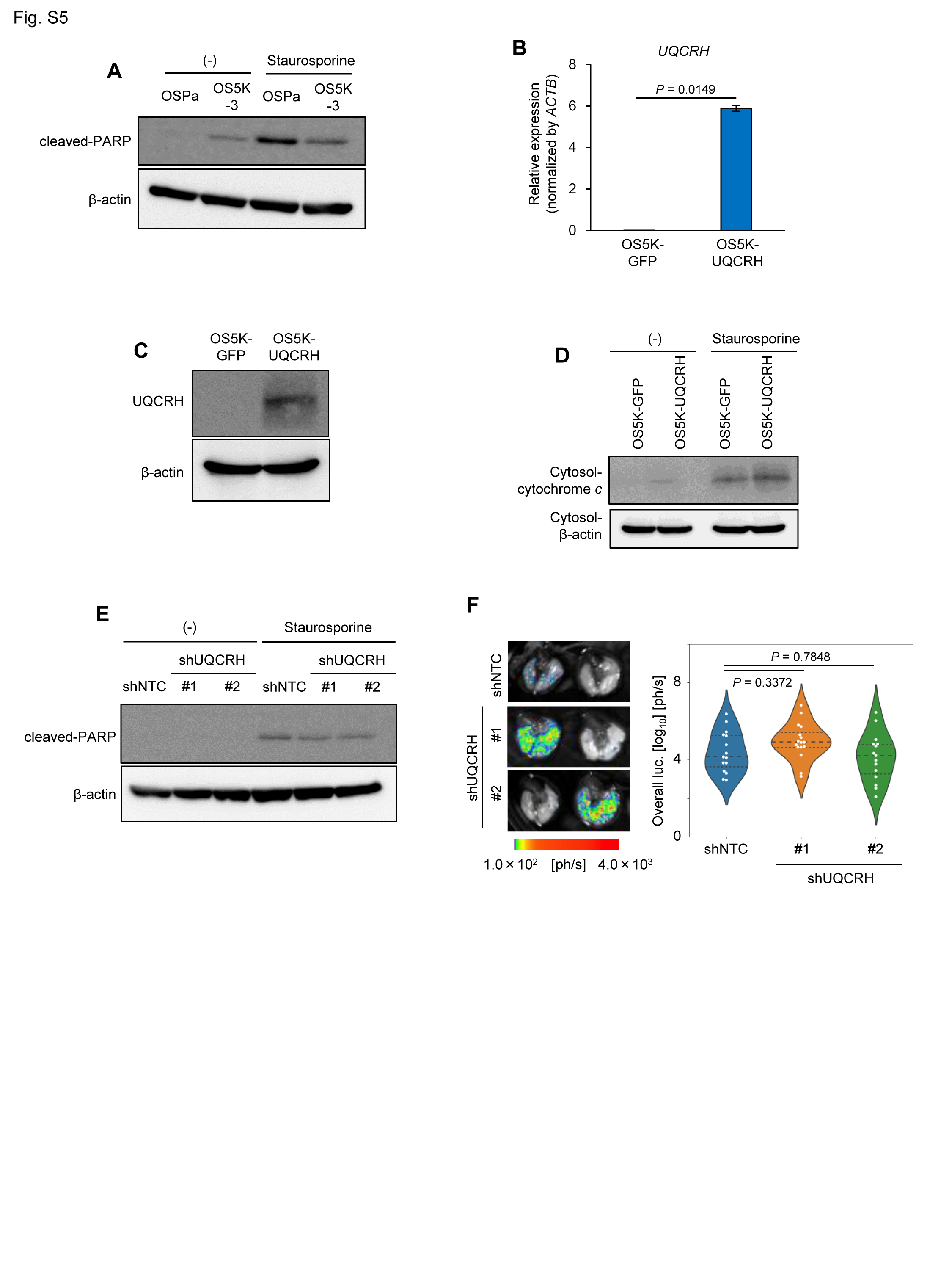

Supplement: Supplementary file 5 — Fig. S5. UQCRH regulates the induction of apoptosis in ccRCC cells. [file MOL2-16-732-s007.tif]

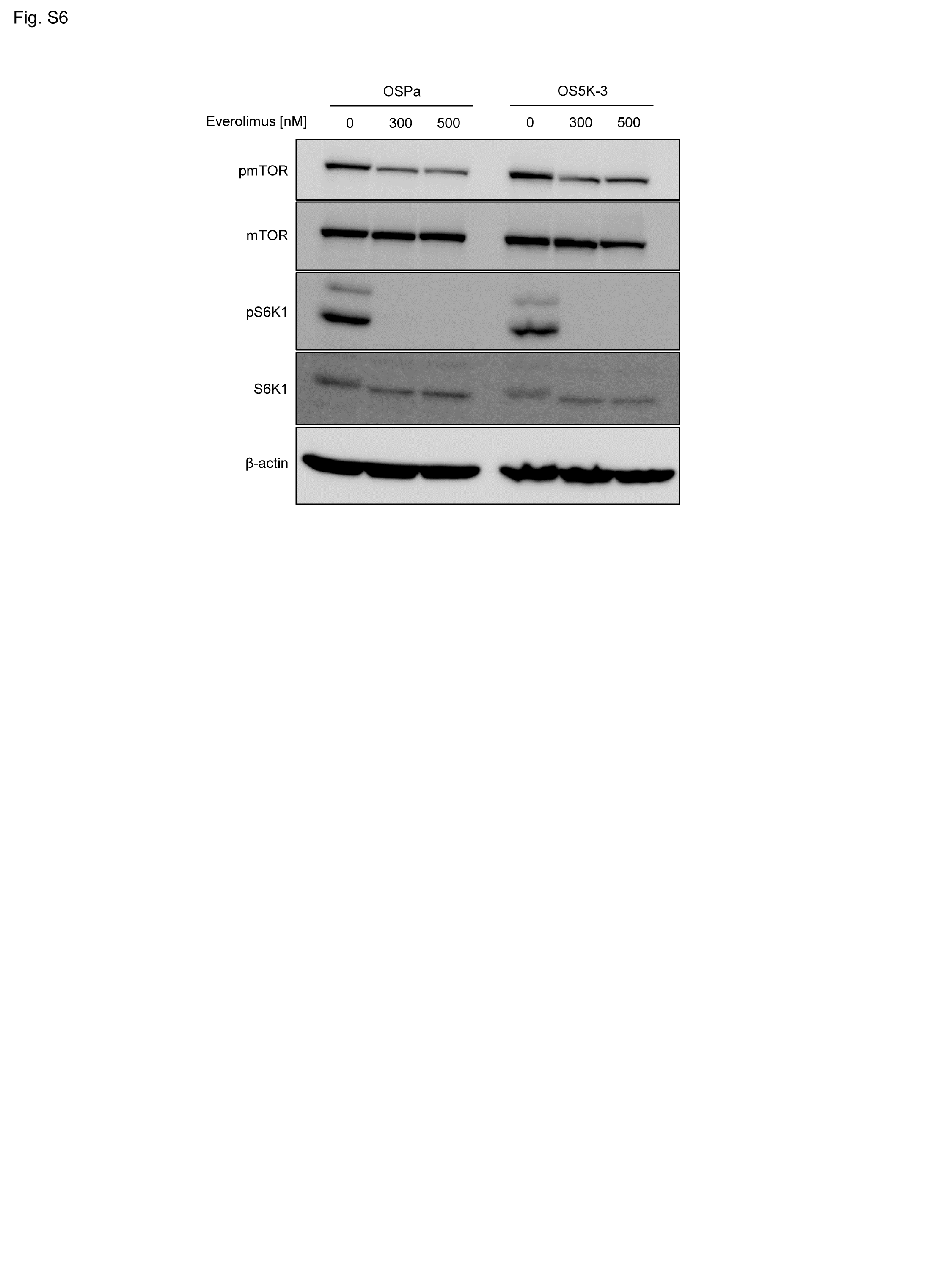

Supplement: Supplementary file 6 — Fig. S6. Inhibition of mTOR signaling in OS‐RC‐2 derivatives by everolimus. [file MOL2-16-732-s005.tif]
