# Supplementary material for: Genome‐wide analysis of DNA methylation identifies the apoptosis‐related gene UQCRH as a tumor suppressor in renal cancer
Source: Mol Oncol. 2021 Jul 5;16(3):732–49. doi: 10.1002/1878-0261.13040 (PMC8807364; doi:10.1002/1878-0261.13040)
Supplement: Supplementary file 7 — Table S1. Target sequences for shRNA. Table S2. Prime sequences for qRT‐PCR analysis. Table S3. Antibodies for immunoblotting. Table S4. Antibodies for immunohistochemistry. Table S5. Primer sequences for bisulfite‐sequencing analysis. [file MOL2-16-732-s001.docx]

**Supporting Information**

**Supplementary Table S1. Target sequences for shRNA.**

| **Target gene** | **shRNA No.** | **Sequence (5' to 3')** |
| --- | --- | --- |
| *UQCRH* | #1 | CGAGCAGTTGGAGAAATGT |
| *UQCRH* | #2 | GCATCAGAATATTTCCTTA |

**Supplementary Table S2. Prime sequences for qRT-PCR analysis.**

| **Gene** | **Orientation** | **Sequence (5' to 3')** |
| --- | --- | --- |
| *ACTB* | Forward | CCTGGCACCCAGCACAAT |
|  | Reverse | GCCGATCCACACGGAGTACT |
| *UQCRH* | Forward | GAGCTCTGTGATGAGCGTGT |
|  | Reverse | CCCTCGCATGCAAGAAGTCA |
| *DNMT3B* | Forward | AGGGAAGACTCGATCCTCGTC |
|  | Reverse | GTGTGTAGCTTAGCAGACTGG |

**Supplementary Table S3. Antibodies for immunoblotting.**

| **First reaction antibody** | **Clone number** | **Catalog number** | **Company** | **Dilution** |
| --- | --- | --- | --- | --- |
| Anti-UQCRH | R40827 | HPA042574 | Sigma-Aldrich | 1/1000 |
| Anti-DNMT3B | 1 | 67259 | Cell Signaling Technology | 1/1000 |
| Anti-β-actin | 127M4857U | A5441 | Sigma-Aldrich | 1/1000 |
| Anti-cleaved-PARP | 15 | 9541 | Cell Signaling Technology | 1/1000 |
| Anti-phospho-p70 S6 kinase | 11 | 9234 | Cell Signaling Technology | 1/1000 |
| Anti-phospho-mTOR | 21 | 2971 | Cell Signaling Technology | 1/1000 |
| Anti-mTOR | 14 | 2983 | Cell Signaling Technology | 1/1000 |
| Anti-p70 S6 kinase | 20 | 9202 | Cell Signaling Technology | 1/1000 |

| **Second reaction antibody** | **Catalog number** | **Company** | **Dilution** |
| --- | --- | --- | --- |
| Anti-rabbit IgG, HRP-linked antibody | 7074 | Cell Signaling Technology | 1/10000 |
| Anti-mouse IgG, HRP-linked antibody | 7076 | Cell Signaling Technology | 1/10000 |

**Supplementary Table S4. Antibodies for immunohistochemistry.**

| **First reaction antibody** | **Clone number** | **Catalog number** | **Company** | **Dilution** |
| --- | --- | --- | --- | --- |
| Anti-UQCRH | R40827 | HPA042574 | Sigma-Aldrich | 1/100 |
| Anti-5-methylcytosine | GR300572-16 | ab10805 | Abcam | 1/400 |

| **Second reaction antibody** | **Catalog number** | **Company** |
| --- | --- | --- |
| Anti-rabbit IgG, HRP-linked antibody | K4003 | Dako |
| Anti-mouse IgG, HRP-linked antibody | K4007 | Dako |

**Supplementary Table S5. Primer sequences for bisulfite-sequencing analysis.**

| **Target gene** | **Orientation** | **Sequence (5' to 3')** |
| --- | --- | --- |
| *UQCRH* | Forward | AAGGGGATGTTTTTTAGTAAAGT |
|  | Reverse | CCTAACCCAATTCAAAATCAAAA |

**Supplementary Fig. S1. Establishment of ccRCC derivatives using serial orthotopic transplantation model.** **A**. Schematic of the experimental procedure. **B**. Cell proliferation assay. Cells were seeded for 3-5 days and counted. The bars represent the mean ± SD (two-sided Student’s *t*-test) **C**. Colony formation assay. Cells were seeded into soft agar and cultured for 15 days. Representative photographs (top). Scale bar, 200 µm. Colony number was quantified (bottom). The bars represent the mean ± SD (two-sided Welch’s *t*-test, *n* = 15) **D**. *In vivo* bioluminescence imaging of the primary tumor. OS-RC-2 derivatives were inoculated into mice and analyzed after 11 days. **E**, **F**. *Ex vivo* bioluminescence imaging of the primary (**E**) and metastatic lung (**F**) tumors in mice in **D**. The mice were sacrificed 17 days after the inoculation. Representative images (left) and their quantification (right). The bars represent the mean and 1st and 3rd quartiles (**E**: two-sided Welch’s *t*-test; **F**: two-sided Student’s *t*-test; *n* = 11, OSPa; *n* = 12 OS5K-3). Figure schematic created with biorender.com.

**Supplementary Fig. S2. Expression and methylation status of genes encoding electron transport chain components in OS-RC-2 derivatives.** Diagram of electron transport chain in mitochondrial inner membrane (top). Expression and methylation status of genes encoding electron transport chain components in OSPa, OS5K-1, OS5K-2, and OS5K-3 cells (bottom). Heatmap shows the reads per kilobase of exon per million mapped reads (RPKM) in each cell in **Fig. 3B** (mRNA) or the β-value difference between the OS5K and OSPa cells in each promoter CpG locus in **Fig. 3A** (methylation status in CpG).

**Supplementary Fig. S3. Clinical significance of UQCRH downregulation in human cancers.** **A**. *UQCRH* mRNA expression in cancer cell lines derived from the indicated organs using the data set from the Broad Institute Cancer Cell Line Encyclopedia. **B**. Decreased UQCRH expression in ccRCC cells in patient-derived xenograft (PDX) models. Data set from the NCBI Gene Expression Omnibus (GEO) database (GSE83820) was analyzed. Blue bars indicate the *UQCRH* mRNA levels in parental ccRCC cells derived from patients. Red bars indicate the *UQCRH* mRNA levels in cells after orthotopic inoculation into mice (*n* = 5).

**Supplementary Fig. S4. Correlation between DNA methylation and UQCRH expression in human cancers.** **A**. Methylation status of the *UQCRH* promoter locus in cancer cell lines derived from the indicated organs. Data set from Broad Institute Cancer Cell Line Encyclopedia was analyzed. **B**. Correlation between UQCRH expression and DNMT expression in ccRCC patients. Data set from TCGA was analyzed. Heatmap shows the expression of UQCRH, DNMT1, DNMT3A, and DNMT3B (left). Correlation analysis of expression of UQCRH and DNMTs (right). The circle size and the number indicate the correlation efficient.

**Supplementary Fig. S5. UQCRH regulates the induction of apoptosis in ccRCC cells.** **A**. Immunoblotting of cleaved-PARP and β-actin in OS-RC-2 derivatives. Cells were treated with staurosporine (50 nM) for 48 h. **B**, **C**. Overexpression of UQCRH in OS5K-3 cells. OS5K-3 cells were infected with lentiviral vectors encoding GFP (OS5K-GFP) or UQCRH (OS5K-UQCRH). *UQCRH* mRNA was measured using qRT-PCR analysis (**B**). The bars represent the mean ± SD (two-sided Welch’s *t*-test, *n* = 2). UQCRH protein was detected using immunoblotting (**C**). **D**. Immunoblotting of cytochrome *c* and β-actin in OS5K-3-GFP and OS5K-3-UQCRH cells. Cells were treated with staurosporine (1 µM) for 6 h. Cytosolic fraction was collected. **E**. Immunoblotting of cleaved-PARP and β-actin in OSPa-shNTC, OSPa-shUQCRH#1, and OSPa-shUQCRH#2 cells. Cells were treated with staurosporine (50 nM) for 48 h. **F**. Representative images (left) of the *ex vivo* bioluminescence imaging of the metastatic lung tumors in mice in **Fig. 6G** and their quantification (right). The bars represent the mean and 1st and 3rd quartiles (one-way ANOVA and Tukey’s test).

**Supplementary Fig. S6.** **Inhibition of mTOR signaling in OS-RC-2 derivatives by everolimus.** Cells were treated with everolimus (0.3 or 0.5 μM) for 2 h. The indicated proteins were detected using immunoblotting.
